# Glu504Lys Single Nucleotide Polymorphism of Aldehyde Dehydrogenase 2 Gene and the Risk of Human Diseases

**DOI:** 10.1155/2015/174050

**Published:** 2015-09-27

**Authors:** Yan Zhao, Chuancai Wang

**Affiliations:** ^1^Department of Bioengineering, Harbin Institute of Technology at Weihai, Shandong 264209, China; ^2^Department of Mathematics, Harbin Institute of Technology at Weihai, Shandong 264209, China

## Abstract

Aldehyde dehydrogenase (ALDH) 2 is a mitochondrial enzyme that is known for its important role in oxidation and detoxification of ethanol metabolite acetaldehyde. ALDH2 also metabolizes other reactive aldehydes such as 4-hydroxy-2-nonenal and acrolein. The Glu504Lys single nucleotide polymorphism (SNP) of *ALDH2* gene, which is found in approximately 40% of the East Asian populations, causes defect in the enzyme activity of ALDH2, leading to alterations in acetaldehyde metabolism and alcohol-induced “flushing” syndrome. Evidence suggests that *ALDH2* Glu504Lys SNP is a potential candidate genetic risk factor for a variety of chronic diseases such as cardiovascular disease, cancer, and late-onset Alzheimer's disease. In addition, the association between *ALDH2* Glu504Lys SNP and the development of these chronic diseases appears to be affected by the interaction between the SNP and lifestyle factors such as alcohol consumption as well as by the presence of other genetic variations.

## 1. Introduction

Aldehyde dehydrogenase (ALDH) 2 is a mitochondrial enzyme that catalyzes the oxidation of acetaldehyde, an intermediate of ethanol metabolism [1]. It is also important in metabolizing other toxic aldehydes such as 4-hydroxy-2-nonenal (4-HNE) and acrolein [2]. The Glu504Lys single nucleotide polymorphism (SNP) of* ALDH2* gene, which occurs with an incidence of 35–57% in different East Asian subpopulations, causes defect in the enzyme activity of ALDH2, leading to alterations in acetaldehyde metabolism and markedly reduced alcohol tolerance [3, 4]. Epidemiological studies have linked* ALDH2* Glu504Lys SNP with increased risk for human diseases including cardiovascular disease (CVD), cancer, and late-onset Alzheimer's disease (AD) [5–8]. The association between* ALDH2* Glu504Lys SNP and the development of these diseases is also related to the effect of the SNP on lifestyle factors such as alcohol consumption and its interaction with other genetic variations.

## 2. ALDH2 in Ethanol Metabolism and Beyond 

ALDH2 is a member of NAD(P)^+^-dependent ALDH supergene family that catalyzes the oxidation of endogenous and exogenous aldehydes to their corresponding carboxylic acids (reviewed in [9]). The enzyme activities of ALDHs mediate the formation of molecules with important biophysiological functions such as retinoic acid, betaine, and gamma-aminobutyric acid [10–12]. On the other hand, aldehydes are highly reactive compounds, which can form adducts with proteins, DNA, and lipids, affecting the function of these biomolecules and leading to cell toxicity. Endogenous aldehydes are generated during the metabolism of amino acids, carbohydrates, lipids, and vitamins as well as the biotransformation of many drugs and environmental chemicals [10, 13–15]. Meanwhile, aldehydes are present in the environment and in our foods. Indeed, aldehydes can be produced in high concentrations by heating fats and sugars (reviewed in [16]). Thus, the detoxification of harmful aldehydes generated endogenously or ingested from environment and foods is an important role of ALDHs.

ALDH2 is best known for its ability to oxidize acetaldehyde, an intermediate of ethanol metabolism. Animal studies have shown that ALDH2 is a major enzyme for acetaldehyde metabolism [1]. In addition, ALDH2 is important for the detoxification of reactive aldehydes such as 4-HNE and acrolein [2]. 4-HNE is an *α*,*β*-unsaturated aldehyde formed during lipid peroxidation* in vivo* [17]. Acrolein is found in small amounts in many foods such as cheese, fish, bread, and alcoholic beverages, while high levels of acrolein can be detected in cigarette smoke and overheated oils (reviewed in [18]). ALDH2 may be one of the essential mechanisms for the removal of these reactive aldehydes and protecting cells and organs from these toxic aldehydes. Furthermore, it has been suggested that ALDH2 may have more than one catalytic function. For example, ALDH2 can act as nitrate reductase, which catalyses the formation of 1,2-glyceryl dinitrate and nitrite from nitroglycerin, leading to the production of cGMP and vasorelaxation [19].

## 3. SNP in* ALDH2* Gene

Human* ALDH2* gene is found on chromosome 12 (12q24.2) and encodes a protein localized in mitochondria matrix. The enzyme is expressed abundantly in liver and is also present in heart, kidney, muscle, and brain [20]. Analyses of the protein structure of ALDH2 have revealed that the enzyme is a tetramer of four identical subunits, each of which is composed of three main domains: the catalytic domain, the NAD^+^-binding coenzyme domain, and the oligomerization domain. A dominant-negative* ALDH2* SNP, which occurs with an incidence of 35–57% in different East Asian subpopulations, results in markedly reduced alcohol tolerance [3, 4]. People carrying the mutant* ALDH2* allele display characteristic acetaldehyde-mediated acute effects of alcohol drinking such as facial flushing and increased pulse rate [21]. The mutation is caused by a single nucleotide substitution of G for A in exon 12, resulting in the replacement of glutamate to lysine at position 504 of the protein [3]. Glu504 is located within the oligomerization domain at the dimer interface of the tetrameric enzyme which is critical for the formation of both dimer and tetramer [22]. In wild-type ALDH2 (ALDH2^*∗*^1), Glu504 forms hydrogen bonds with Arg281 of the same subunit and with Arg492 of the adjacent dimer partner. The disruption of these interactions by the presence of Lys504 in the mutant (ALDH2^*∗*^2) perturbs the structure of the subunit with the mutation as well as its dimer partner [23]. Binding of the coenzyme NAD^+^ to ALDH2^*∗*^2 is impaired, and the mutant enzyme has an increased *K*_*m*_ for NAD^+^ and a decreased *k*_cat_, which leads to a very low enzymatic activity* in vivo* [24]. Therefore, ALDH2^*∗*^2 acts in a dominant-negative manner. The phenotypic loss of ALDH2 activity is found in both heterozygous (*ALDH2* ^*∗*^ *1/* ^*∗*^ *2*) and homozygous (*ALDH* ^*∗*^ *2/* ^*∗*^ *2*) individuals, whose blood acetaldehyde concentrations are approximately 6 and 19 times higher than those with active ALDH2, respectively, following a low to moderate intake of alcohol [25].

## 4. Glu504Lys SNP of* ALDH2* Gene and the Risk of Human Diseases

The high blood acetaldehyde levels in individuals with* ALDH2* ^*∗*^ *2* allele after moderate alcohol consumption mediate the enhanced alcohol sensitivity in this population [26]. In comparison to* ALDH2* ^*∗*^ *1/* ^*∗*^ *1* homozygotes, individuals with* ALDH2* ^*∗*^ *1/* ^*∗*^ *2* genotype experience significantly higher pulse rate and greater facial flushing as early as 30 min following alcohol consumption and have higher risk of developing hangover symptoms [21, 27]. Electroencephalographs (ECG) show that the increases in P300 latency and decreases in P300 amplitude following alcohol consumption are greater in individuals with* ALDH2* ^*∗*^ *1/* ^*∗*^ *2* genotype than in individuals with* ALDH2* ^*∗*^ *1/* ^*∗*^ *1* genotype, suggesting that their cognitive functioning may be more impaired by alcohol exposure [28, 29]. Individuals with* ALDH2* ^*∗*^ *1/* ^*∗*^ *2* genotype also show more impaired psychomotor performance including reaction time to complex visual information, visuomotor coordination, and exact motor ability than those with* ALDH2* ^*∗*^ *1/* ^*∗*^ *1* at 30 and 60 min after moderate alcohol consumption [29, 30]. Because of these acute effects after alcohol ingestion, Glu504Lys SNP of* ALDH2* gene is protective against the development of alcoholism and perhaps may decrease the risk of chronic diseases caused by alcohol overconsumption. On the other hand, individuals carrying Glu504Lys SNP who do drink alcohol may have an increased incidence of alcohol-mediated diseases. Furthermore, for susceptible individuals that drink limited amount of alcohol, defective ALDH2 may cause the accumulation of toxic aldehydes, which can be generated endogenously from metabolism other than ethanol oxidation or directly enter the body from foods and the environment, leading to enhanced oxidative stress [31] and impaired cell function and subsequently affecting the risk of a variety of human chronic diseases.

### 4.1. CVD and Associated Diseases

Although liver is considered as the primary site for ethanol oxidation, other organs such as heart also participate in ethanol metabolism. Elevated acetaldehyde can lead to cardiac toxicity and plays a significant role in the pathogenesis of alcoholic cardiomyopathy (reviewed in [32]). Overexpression of the* ALDH2* gene alleviates oxidative stress and apoptosis induced by ethanol and acetaldehyde in human cardiac myocytes [33] and mitigates mechanical anomalies induced by alcohol in myocardium of* ALDH2* transgenic mice [34], suggesting that the detoxification of acetaldehyde by ALDH2 protects against alcohol-induced cardiac toxicity. In addition, accumulation of cytotoxic aldehydes other than acetaldehyde, which are either generated during reactive oxygen species (ROS)-induced stress or ingested from foods or polluted environment, such as 4-HNE and acrolein, contributes to the oxidative stress and impairs cardiac functions [35–37]. 4-HNE can be endogenously produced from lipid peroxidation of polyunsaturated fatty acids in conditions like ischemia and reperfusion of heart. In fact, accumulation of 4-HNE-protein adducts and protein carbonyls has been detected in the failing hearts [38] and has been shown to depress contractility of isolated cardiac myocytes, inhibit mitochondrial function, and cause tissue damage after cardiac ischemia [39, 40]. Another toxic aldehyde acrolein is present in a variety of foods and is detected in high levels in cigarette smoke and overheated oils (reviewed in [18]). In a mouse model of acute myocardial infarction, dietary treatment with acrolein at concentration (5 mg/kg) comparable to human diet, 24 h prior to a 30 min coronary artery occlusion and 24 h reperfusion, significantly increases myocardial infarct size, exacerbating cardiac injury caused by ischemia and reperfusion [41]. Acrolein also blocks cardioprotective effects induced by the pretreatment of NO donor diethylenetriamine/NO via mechanisms that disrupt protein kinase C*ε* signal transduction [41]. Thus, detoxification of toxic acetaldehydes such as those mentioned above by ALDH2 could be beneficial for prevention and intervention of CVD. In a rat model where heart failure is induced by a 6-week treatment of myocardial infarction, treatment of a selective ALDH2 activator Alda-1, starting 4 weeks after myocardial infarction, significantly decreases the accumulation of 4-HNE and its associated cell toxicity in failing hearts and improves cardiomyocyte shortening, left ventricular compliance, and diastolic function [38]. Consistently, overexpression of* ALDH2* gene in mice decreases 4-HNE levels elevated by ischemia and reperfusion and significantly alleviates ischemia/reperfusion injury and hypoxia/reoxygenation-induced cardiomyocyte contractile dysfunction. In contrast, the accumulation of cardiac 4-HNE and the cardiac injury in response to ischemia-reperfusion are exacerbated in* ALDH2* knockout mice [42].

These findings suggest that disruption of ALDH2 activity may increase the susceptibility of an individual to CVD. Recently, two meta-analyses have shown that Glu504Lys SNP of* ALDH2* gene in Asian populations is associated with increased risk of coronary artery disease (odds ratio (OR) = 1.36 and 1.28, 95% confidence interval (CI) = 1.06–1.75 and 1.10–1.48, and *p* = 0.017 and 0.001, resp.) and myocardial infarction (OR = 1.64 and 1.58, 95% CI = 1.22–2.20 and 1.15–2.19, and *p* = 0.001 and 0.005, resp.) [6, 7]. It has been reported that* ALDH2* ^*∗*^ *2* allele is associated with low serum HDL cholesterol levels in Asian populations [43]. In addition, as ALDH2 also functions in the formation of nitric oxide from nitroglycerin, Glu504Lys SNP of* ALDH2* gene eliminates the activity of the enzyme to catalyze the reaction. Therefore, it is not surprising that* ALDH2* ^*∗*^ *2* allele is associated with a lack of an efficacious clinical response to nitroglycerin treatment for coronary heart disease [44].

Surprisingly,* ALDH2* ^*∗*^ *2* carriers with chronic cyanosis are shown to have unexpectedly greater tolerance to ischaemia and reperfusion injury in comparison to* ALDH2* ^*∗*^ *1* homozygotes.* ALDH2* ^*∗*^ *2* carriers have lower postoperative troponin I levels and inotropic scores as well as shorter length of intensive care unit (ICU) and hospital stay after open-heart surgery [45]. It has been found that aldehyde accumulation caused by cyanosis in* ALDH2* ^*∗*^ *2* carriers results in larger myocardium glutathione (GSH) pools [45]. The increased intracellular GSH levels are also seen in the hearts of* ALDH2* ^*∗*^ *2* transgenic mice when compared with those of wild-type controls [46]. This compensatory myocardium GSH pool may contribute to the unexpectedly better cardioprotection seen in the* ALDH2* ^*∗*^ *2* patients [45].

### 4.2. Hypertension

Hypertension is a major risk factor for CVD. It is known that excessive alcohol consumption promotes the development of hypertension. The effects of Glu504Lys SNP of* ALDH2* gene on blood pressure are complicated by alcohol consumption and the presence of other genetic polymorphisms. It has been found that the prevalence of hypertension is higher in* ALDH2* ^*∗*^ *1* homozygotes (OR = 1.67, 95% CI = 1.37–2.08, and *p* < 0.0001) in comparison to* ALDH2* ^*∗*^ *2* carriers among males in a Japanese population. Further investigation on* ALDH2* genotypes and the level of alcohol consumption suggests that the* ALDH2* ^*∗*^ *1/* ^*∗*^ *1* genotype correlates with increased risk for hypertension among males primarily through its association with the level of alcohol consumption [47]. Similar results have been reported by Amamoto et al. The authors have found that* ALDH2* ^*∗*^ *2* carriers have lower incidence of hypertension than* ALDH2* ^*∗*^ *2* noncarriers (OR = 0.67, 95% CI = 0.47–0.96, *p* = 0.030), while this correlation is not observed in individuals whose alcohol consumption is below median level or in the group not taking antihypertensive agents [48]. A case-control study later has shown that* ALDH2* ^*∗*^ *1/* ^*∗*^ *1* genotype is an independent risk factor for essential hypertension among males. The OR for the presence of hypertension for* ALDH2* ^*∗*^ *1/* ^*∗*^ *1* genotype compared with other genotypes is 1.93 (95% CI = 1.12–3.31, *p* = 0.018) [49]. In Japanese male regular drinkers (≥22 g ethanol/d), Tsuchihashi-Makaya et al. have reported that* ALDH2* ^*∗*^ *1/* ^*∗*^ *1* genotype is an independent predictor for increased systolic (*β*-coefficient = 2.96, *p* = 0.03) and diastolic (*β*-coefficient = 2.26, *p* = 0.01) blood pressure after adjusting for alcohol consumption [50]. More recently, it has also been observed in a Chinese Han population that* ALDH2* ^*∗*^ *2* carriers who drink alcohol have lower risk of essential hypertension (OR = 0.55, 95% CI = 0.36–0.85), while this association is not found in nondrinkers [51]. It is proposed that the vasodilating effect of acetaldehyde [52] may be one of the mechanisms for the lower blood pressure seen in the* ALDH2* ^*∗*^ *2* carriers who drink alcohol [50].

Conversely, Chang et al. have reported that* ALDH2* ^*∗*^ *2* allele is significantly associated with increases of blood pressure (systolic blood pressure: 0.865 mmHg/yr, diastolic blood pressure: 0.537 mmHg/yr) in a prospective Chinese cohort followed on an average of 5.7 yrs [53]. The authors suggest that carriers with* ALDH2* ^*∗*^ *2* allele may be more susceptible to progress to hypertension compared with noncarriers [53]. The size and characteristics (age, gender distribution, eating habits, average amount of alcohol consumption, medications, etc.) of the population examined may cause the inconsistent results seen in different studies. A total of 753 individuals from 276 families are included in the follow-up study mentioned above [53]; it is possible that other genetic polymorphisms may exist in this population and act together with* ALDH2* genotype in combination with dietary/environmental factors to promote hypertension. Indeed, the benefit of* ALDH2* ^*∗*^ *2* allele on blood pressure has been shown to disappear when the effect is evaluated in combination with* SOD2* polymorphism. Individuals carrying both* ALDH2* ^*∗*^ *2* allele and* SOD2* Val/Val genotype have a significantly higher risk of hypertension among drinkers than in nondrinkers (adjusted OR = 6.22, 95% CI = 2.26–17.1, and *p* < 0.001) [54]. Therefore, both lifestyle factors such as alcohol drinking and other genetic variations may have impacts on the susceptibility of* ALDH2* genotypes to hypertension.

### 4.3. Cancer

Aldehydes are very reactive molecules, which can modify proteins and nucleic acids, causing dysfunction of these biomolecules. Binding of acetaldehyde with DNA has been demonstrated to promote carcinogenesis in laboratory animals and alcoholic individuals [55, 56]. Indeed, chronic alcohol consumption has been shown to be a strong risk factor for the cancer of many tissues and organs including the upper aerodigestive tract (oral cavity, pharynx, larynx, and oesophagus), liver, colorectum, and breast [57, 58]. Acetaldehyde is considered to be one of the important mechanisms contributing to the development of alcohol-associated cancers [55, 56].

The effect of the alteration of ALDH2 activity by Glu504Lys SNP on the risk of cancer has been shown to interact with lifestyle factors, especially alcohol consumption. Yokoyama et al. have reported that, in male Japanese alcoholics, the* ALDH2* ^*∗*^ *2* allele significantly increases the risks (OR) for the oro-pharyngo-laryngeal (11.14), esophageal (12.50), stomach (3.49), colon (3.35), and lung (8.20) cancer, but not for liver or other cancers after adjustment for age, daily alcohol consumption, and amount of cigarette smoking [8]. Further evaluation of the association of* ALDH2* Glu504Lys SNP with esophageal cancer has demonstrated that OR for the* ALDH2* ^*∗*^ *1/* ^*∗*^ *2* and* ALDH2* ^*∗*^ *2/* ^*∗*^ *2* genotypes in comparison to the* ALDH2* ^*∗*^ *1/* ^*∗*^ *1* genotype is 3.43 (95% CI = 1.74–6.75) after adjustment for age, sex, drinking, and smoking status. A strong gene-environment interaction exists between* ALDH2* ^*∗*^ *2* allele and excessive alcohol consumption for the risk of esophageal cancer. The OR for heavy drinkers with* ALDH2* ^*∗*^ *2* relative to nonheavy drinkers with* ALDH2* ^*∗*^ *1/* ^*∗*^ *1* genotype is 6.84 (95% CI = 2.39–19.6) [59]. A case-control study on patients with esophageal squamous cell carcinoma from Taiwan has shown similar results. In the study, it has been found that individuals with* ALDH2* ^*∗*^ *1/* ^*∗*^ *2* and* ALDH2* ^*∗*^ *2/* ^*∗*^ *2* genotypes have 4.99- (95% CI = 3.11–7.99) and 4.24-fold (95% CI = 1.52–11.84) risk, respectively, of developing esophageal cancer, when compared with those with* ALDH2* ^*∗*^ *1/* ^*∗*^ *1* genotype, after adjustment for appropriate covariates. And the heavy drinkers (≥1,200 g/year) with* ALDH2* ^*∗*^ *1/* ^*∗*^ *2* genotype have 30.53-fold risk (95% CI = 12.01–77.64) of developing esophageal cancer in comparison to nondrinkers with* ALDH2* ^*∗*^ *1/* ^*∗*^ *1* [60].

*ALDH2* Glu504Lys polymorphism has also been shown to interact with the SNPs of other key enzymes in ethanol metabolism in the development of alcohol-associated cancers [61]. Alcohol dehydrogenase 1B (ADH1B) is one of the major enzymes belonging to a group of ADHs that break down ethanol to acetaldehyde. Arg47His SNP of* ADH1B* (*ADH1B* ^*∗*^ *2*), which exists in more than 90% of East Asians, encodes a superactive subunit of ADH1B that promotes the accumulation of acetaldehyde after alcohol drinking [61]. In Japanese alcoholic patients, blood levels of N(2)-ethylidene-2′-deoxyguanosine (N(2)-ethylidene-dG), the most abundant acetaldehyde-derived DNA adduct, are remarkably higher in individuals carrying both* ALDH2* ^*∗*^ *2* and* ADH1B* ^*∗*^ *2* alleles, suggesting that alcoholic individuals with both SNPs may accumulate more DNA damage and may have increased susceptibility to cancer development [62]. Surprisingly, a study on Japanese alcoholic men (age > 40 y) has shown that individuals with* ADH1B* ^*∗*^ *1/* ^*∗*^ *1* genotype (OR = 2.03) have increased risk of esophageal cancer after adjustment for drinking and smoking.* ALDH2* ^*∗*^ *1/* ^*∗*^ *2* genotype also had higher risk of esophageal cancer (OR = 12.76). For individuals with* ALDH2* ^*∗*^ *1/* ^*∗*^ *2* and* ADH1B* ^*∗*^ *1/* ^*∗*^ *1* genotypes, the esophageal cancer risk is enhanced in a multiplicative fashion (OR = 27.66) [63]. A few mechanisms are proposed for this unexpected result. First, it has been demonstrated that the lower systemic elimination of ethanol from the body by* ADH1B* ^*∗*^ *1/* ^*∗*^ *1* may lead to increased production of acetaldehyde by oral microbes, thus prolonging the exposure to acetaldehyde through saliva [64]. In addition, individuals who carry the highly active* ADH1B* ^*∗*^ *2* allele rapidly convert ethanol to acetaldehyde following alcohol consumption, leading to the accumulation of acetaldehyde and the facial flushing syndrome. The unpleasant symptoms prevent* ADH1B* ^*∗*^ *2* carriers from drinking alcohol, therefore perhaps exerting a protective effect against alcohol-associated cancer development [57].

Recently, studies have confirmed that the* ALDH2* ^*∗*^ *2* allele is associated with an increased risk of gastric cancer [65, 66]. It has also been found that there is an interaction between* ALDH2* Glu504Lys SNP and alcohol consumption in the development of gastric cancer [65]. The studies on the association of the* ALDH2* Glu504Lys SNP with the risk of colorectal cancer have given inconsistent results. Two recent meta-analyses indicate that* ALDH2* Glu504Lys SNP may be associated with a decreased risk of colorectal cancer [67, 68]. No significant impact of Glu504Lys SNP of* ALDH2* gene on the risk of hepatocellular carcinoma [69] and breast cancer has been found in East Asian populations [70, 71].

### 4.4. Alzheimer's Disease

AD is the most common neurodegenerative disease that causes dementia in the elderly. The major pathological characteristics of AD brains are the presence of senile plaques composed of beta-amyloid peptide (Abeta), neurofibrillary tangles (NFT) formed by hyperphosphorylated tau protein, and neuronal loss (reviewed in [72, 73]). Accumulating evidence has shown that oxidative stress is one of the important factors in the pathogenesis of AD (reviewed in [74]). Products of lipid peroxidation such as 4-HNE have been reported to be elevated in the brains of AD patients [75]. It has been found that 4-HNE can induce neuronal death and synapse dysfunction [76] and markedly inhibit microtubule formation and neurite outgrowth [77]. 4-HNE has also been shown to react with phosphorylated tau and induce conformational changes in tau proteins that promote the formation of NFT [78, 79]. In addition, exposure of NT(2) neurons to 4-HNE elicits an upregulation of the expression of beta-site amyloid precursor protein cleaving enzyme (BACE), causing significant increase in intracellular and secreted levels of Abeta peptides [80]. Thus, elevated levels of 4-HNE in central nervous system may contribute to the pathogenesis of AD. In PC12 cells, ALDH2 deficiency produced by introducing* ALDH2* ^*∗*^ *2* gene leads to marked accumulation of 4-HNE in response to oxidative stress stimuli and increased vulnerability to 4-HNE-induced cell death [81]. Similarly, central neurons from transgenic mice overexpressing* ALDH2* ^*∗*^ *2* gene are more sensitive to 4-HNE-induced toxicity than cells from control animals [82]. Moreover, these ALDH2 deficient mice exhibit an age-dependent decrease in spatial cognitive ability starting at the age of 6 months [82]. The reduced resistance to oxidative stress has been proposed as one mechanism that leads to the neurodegeneration and memory loss in ALDH2 deficient mice [82]. These results suggest that* ALDH2* Glu504Lys SNP could be a genetic risk factor for AD in susceptible populations.

A case-control study from Japan has shown that the number of individuals carrying* ALDH2* ^*∗*^ *2* allele is significantly higher in the patients with late-onset AD (LOAD) than in the controls (48.1% versus 37.4%, *p* = 0.001) [5].* ALDH2* ^*∗*^ *2* genotype interacts synergistically with the presence of the* apolipoprotein E allele 4* (*APOE-ε4*), which is a widely accepted risk factor for LOAD [5]. Logistic regression analysis shows that* ALDH2* ^*∗*^ *2* allele increases the risk for LOAD independently of* APOE-ε4* (*p* = 0.002) status, while the coexistence of the* APOE-ε4* allele and* ALDH2* ^*∗*^ *2* allele synergistically increases the frequency of LOAD, which is 31 times higher in individuals being* APOE-ε4* homozygous and having at least one* ALDH2* ^*∗*^ *2* allele than in those having neither allele [5]. Moreover, in LOAD patients homozygous for* APOE-ε4*, the age at onset of LOAD is significantly younger in those with* ALDH2* ^*∗*^ *2* allele than in those without* ALDH2* ^*∗*^ *2* allele, and the dosage of the* ALDH2* ^*∗*^ *2* allele significantly affects the age at onset of the disease [5]. Interestingly, immunostaining of the brain of AD patients using anti-4-HNE antibody reveals that the cytoplasm of pyramidal cell is positive for 4-HNE only in individuals with* APOE-ε4* allele [83]. Studies have shown that APOE proteins interact with 4-HNE and the strength of binding between APOE isoforms and 4-HNE is different, with the order *ε*2 > *ε*3 > *ε*4. This correlates with the differential protective effect of APOE isoforms against 4-HNE-induced neuronal apoptosis [84]. These results suggest that APOE may have an important role in elimination of 4-HNE. And the possession of APOE-*ε*4, the APOE with the weakest 4-HNE binding ability, may lead to the accumulation of toxic 4-HNE in neurons, which could be further intensified by the reduction of ALDH2 activity, resulting in increased oxidative stress and higher risk for developing AD [82].

It has been observed in a Chinese case-control study that individuals carrying* ALDH2* ^*∗*^ *2* allele have significantly higher risk of AD (OR = 3.11, 95% CI = 2.06–4.69, and *p* < 0.001) [85]. In contrast, a study on sporadic AD patients from Mongolic ethnic group in China has not found an association between* ALDH2* ^*∗*^ *2* allele and the risk of AD [86]. There may be several reasons underlying the discrepancies found in these studies. First, the populations analyzed are from different ethnic backgrounds or different geographic regions; thus it is not impossible that other genetic polymorphisms may have an impact on the results. Secondly, environmental factors such as eating habits or alcohol consumption of the studied subjects should be considered and may influence the results. In addition, the sample size as well as the distribution of male/female subjects in the studies may affect the significance of the result. Recently, a meta-analysis evaluated the association of* ALDH2* variants with the risk of AD in East Asian populations has found that* ALDH2* ^*∗*^ *1/* ^*∗*^ *2* and ^*∗*^ *2/* ^*∗*^ *2* genotypes are associated with increased AD risk only in subgroup analyses in which male subjects are included (OR = 1.72, 95% CI = 1.10–2.67, and *p* = 0.02) [87].

## 5. Conclusions

**Figure 1:**
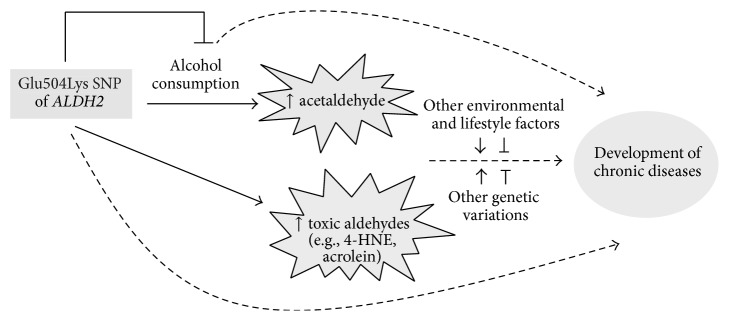
The association of Glu504Lys SNP of* ALDH2* gene with human diseases is complicated by other genetic variations and environmental/lifestyle factors in addition to alcohol drinking.

ALDH2 is the major enzyme for the clearance of ethanol metabolite acetaldehyde. It is also important for our body to metabolize other toxic aldehydes, such as lipid peroxidation product 4-HNE, generated endogenously or ingested from environment. Dysfunction of ALDH2 in individuals carrying* ALDH2* Glu504Lys SNP leads to increased accumulation of toxic aldehydes that may result in higher risk of a variety of human diseases including CVD, cancer, and AD. Here we discuss the evidence that implicates* ALDH2* ^*∗*^ *2* allele as a candidate genetic risk factor for these chronic diseases. It has to be noted that the pathogenesis of these chronic diseases involves multiple mechanisms; thus the interactions between* ALDH2* ^*∗*^ *2* allele and other genetic polymorphisms as well as the gene-environment interactions have to be considered to give a more thorough picture on how* ALDH2* genotype affects the development of chronic diseases. In particular, the Glu504Lys SNP of* ALDH2* gene is closely related to alcohol drinking behavior. For alcoholics,* ALDH2* ^*∗*^ *2* allele may significantly exacerbate the risk of alcohol-related health problems. On the other hand, the unpleasant flushing syndromes caused by the accumulation of acetaldehyde result in less alcohol consumption and reduced incidence of alcoholism in individuals carrying* ALDH2* ^*∗*^ *2* allele. As a consequence, for pathogenesis directly associated with ethanol consumption, Glu504Lys SNP of* ALDH2* gene can be a protective factor for* ALDH2* ^*∗*^ *2* allele carriers who limit their exposure to ethanol. In addition, the effect of Glu504Lys SNP of* ALDH2* gene on the development of chronic diseases, which are often multifactorial, can be complicated by other genetic variations [54]. In summary, defective ALDH2 activity may compromise the elimination of reactive aldehydes, leading to increased cytotoxicity and oxidative stress. Other genetic or environmental/life style factors, which promote (or inhibit) the stress caused by defective ALDH2, may increase (or reduce) the susceptibility of individuals carrying* ALDH2* ^*∗*^ *2* allele to relevant chronic diseases (Figure 1).

More effort should be spent in the future to understand the biochemical and molecular mechanisms underlying the association of* ALDH2* Glu504Lys SNP with chronic diseases. Studies using animal and cell models with defective ALDH2 activity will further our knowledge on the molecular basis of human phenotype of* ALDH2* variant and provide information for discovery of potential interventions and therapeutics targeting ALDH2.

Finally, for those diseases that are prompted synergistically by* ALDH2* Glu504Lys SNP and alcohol consumption, individuals carrying* ALDH2* ^*∗*^ *2* allele should be targeted for reducing alcohol exposure as part of the preventive strategy.
